# Assessing the impact of curcumin on dual‐species biofilms formed by *Streptococcus mutans* and *Candida albicans*

**DOI:** 10.1002/mbo3.937

**Published:** 2019-09-27

**Authors:** Xinlong Li, Luoping Yin, Gordon Ramage, Bingchun Li, Ye Tao, Qinghui Zhi, Huancai Lin, Yan Zhou

**Affiliations:** ^1^ Department of Preventive Dentistry Guanghua School of Stomatology Sun Yat‐sen University Guangzhou China; ^2^ Guangdong Provincial Key Laboratory of Stomatology Sun Yat‐sen University Guangzhou China; ^3^ Oral Sciences Research Group Glasgow Dental School School of Medicine, Dentistry and Nursing College of Medical, Veterinary and Life Sciences University of Glasgow Glasgow UK

**Keywords:** biofilm, *Candida albicans*, curcumin, *Streptococcus mutans*

## Abstract

*Streptococcus mutans* and *Candida albicans* are often isolated from plaques associated with early childhood caries. However, there are limited studies examining how these microorganisms interact with one another and how best to manage them. Recent studies have shown that curcumin (CUR), a natural compound, has the potential to independently control both of these microorganisms. The purpose of this study was to investigate how *S. mutans* and *C. albicans* respond in mono‐ and dual‐species biofilms challenged with CUR. Quantitative biofilm biomass and viability were first evaluated and supported by live–dead PCR to assess biofilm composition. Confocal laser scanning microscopy (CLSM) was used to evaluate the exopolysaccharide (EPS) content and thickness of the biofilms, and the structure of the biofilms and morphology of the cells were observed by scanning electron microscopy (SEM). Quantitative real‐time PCR (qRT‐PCR) was applied to assess relative gene expression. The 50% minimum biofilm eradication concentration (MBEC_50_) of CUR against *S. mutans* and *C. albicans* was 0.5 mM. The biomass and viability decreased after treatment with CUR both in dual‐species biofilms and in mono‐species biofilm. CUR inhibited *S. mutans* and *C. albicans* in both mono‐ and dual‐species biofilms. *Streptococcus mutans* was more sensitive to CUR in dual‐species biofilm than in mono‐species biofilms, whereas *C. albicans* was less sensitive in dual‐species biofilms. EPS production was decreased by CUR in both mono‐ and dual‐species biofilms, which coincided with the downregulation of glucosyltransferase and quorum sensing‐related gene expression of *S. mutans*. In *C. albicans,* the agglutinin‐like sequence family of *C. albicans* was also downregulated in dual‐species biofilms. **C**ollectively, these data show the potential benefit of using a natural antimicrobial, CUR, to control caries‐related dual‐species plaque biofilms.

## Graphical abstract

Curcumin, a natural compound, can inhibit oral dual‐species biofilms. The results here show that curcumin has not only inhibited the mono‐species biofilms from *Streptococcus mutans* and *Candida albicans*, but also the dual‐species biofilm formed by those two species. Exopolysaccharide production was decreased by curcumin treatment in both mono‐ and dual‐species biofilms.

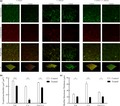

## 1 INTRODUCTION

Polymicrobial infection occurs during the process of dental caries development. *Streptococcus mutans* interacts with *Candida albicans* to form cross‐kingdom biofilms associated with early childhood caries (ECC) (Hajishengallis, Parsaei, Klein, & Koo, 2017). The amount of *C. albicans* is dramatically higher in children with ECC than in those without tooth decay (Ghasempour, Sefidgar, Eyzadian, & Gharakhani, 2011). Studies have shown that the presence of *C. albicans* can augment exopolysaccharides (EPS) in biofilms, resulting in an increase in biomass related to mono‐species biofilms of *S. mutans* alone (Falsetta et al., 2014; Hwang et al., 2017). *S. mutans*–*C. albicans* associations enhance *S. mutans* infection, modulate the structure of biofilms, and thus influence the onset and severity of dental caries (Falsetta et al., 2014).

Eradicating these bacterial–fungal biofilms in vitro is challenging (Heitman, Metwalli, Khan, Krom, & Jabra‐Rizk, 2013). Most of the clinically used therapeutic approaches are monotherapies, based on either antibacterial or antifungal agents despite the polymicrobial nature of disease‐causing biofilms (Xiao et al., 2018). Fluoride is widely used in the prevention of dental caries. However, fluoride does not offer protection against the infectious aspects and instead is involved in the remineralization process (Ten Cate, 2012). The long‐term use of chlorhexidine will lead to staining of the tongue and taste disorders (Neturi, 2014; Rahmani‐Badi, Sepehr, & Babaie‐Naiej, 2015). Antibiotic resistance also limits the application of therapeutic approaches to control biofilm infection (Marc et al., 2018). Finding new therapeutic methods for disrupting bacterial–fungal interkingdom biofilms could help improve anticaries strategies and oral health. Naturally derived chemotherapeutic agents are an attractive option. They have advantages over synthetic derivatives due to their natural evolution and diminished likelihood of resistance. Curcumin (CUR), a food‐grade natural product extracted from the root of turmeric, is widely used as a flavoring and coloring agent in food. It is also used in clinical practice due to its anti‐inflammatory and antitumor activities (Esatbeyoglu et al., 2012). Our group has shown that CUR exerts promising anticaries effects on *S. mutans* by altering the EPS synthesis mechanism (Li, Li, Lin, & Zhou, 2018).

Since dual‐species biofilms of *S. mutans* and *C. albicans* have the advantage of better simulating the pathogenic biofilms in ECC relative to mono‐species biofilms, exploration of the effect of CUR on dual‐species biofilms should be prioritized. In this study, we establish an in vitro biofilm model by using *S. mutans* and *C. albicans*, and investigate the reaction of dual‐species biofilms to CUR.

## 2 MATERIALS AND METHODS

### 2.1 Growth conditions and MBEC assay to determine the effects of CUR on *S. mutans* and* C. albicans*

*Streptococcus mutans* UA159 (ATCC 700610) and *C. albicans* SC5314 (ATCC MYA‐2876) were selected for this study. *Candida albicans* was grown in Sabouraud's dextrose broth (SDB, HKM) at 37°C with shaking at 200 rpm for 18 hr. *Streptococcus mutans* was cultured in brain heart infusion broth (BHI, Difco) for 18 hr at 37°C. CUR was dissolved in dimethyl sulfoxide (DMSO) for liquid storage.

*Streptococcus mutans* and *C. albicans* were cultured in BHI and SDB separately for 18 hr. Cellular microorganisms were collected by centrifugation (13,201 *g*, 5 min, 4°C). Phosphate‐buffered saline (PBS) was applied to wash the cultures three times. Suspensions of microorganisms were standardized to OD values equal to 1 × 10^7^ CFUs/ml using a microplate spectrophotometer (Infinite 200, TECAN). Biofilms were grown in 96‐well flat‐bottom plates. The minimum biofilm eradication concentration (MBEC) for *S. mutans* and *C. albicans* biofilms was determined by the colony counting method. The biofilms were cultured following the methodology described above for 24 hr in 96‐well flat‐bottom plates. The medium was refreshed, and serial dilutions of CUR were added to the biofilms. Medium with the corresponding concentrations of DMSO without CUR was used as a control. After 24 hr of cultivation, the supernatants were removed, and sterile PBS was gently applied three times to wash the biofilms that formed on the plates. The biofilms were scraped and then resuspended in PBS, and the CFU counts were used to confirm the MBEC of the microorganisms.

### 2.2 The effects of CUR on mono‐ or dual‐species biofilms in vitro

The biofilms were grown as described in previous studies with some modification (Willems, Kos, Jabra‐Rizk, Krom, & Bjarnsholt, 2016; Zhou et al., 2018). The cells were cultured as previously described and standardized by artificial saliva solution (ASS, NOVON) supplemented with 1% sucrose and 10% fetal bovine serum to OD_600_ = 0.1. Three test groups with CUR (*S. mutans* biofilm, *C. albicans* biofilm, and *S. mutans‐C. albicans* biofilm) and corresponding control groups without CUR were established.

The biofilms formed on 6‐well flat‐bottom microtiter plates (Coster Corning). For the dual‐species biofilms, 1.5 ml of *S. mutans* and 1.5 ml of *C. albicans* were added to the microtiter plate. To form mono‐species biofilms, 3 ml of *S. mutans* and 3 ml of *C. albicans* were added separately. The plates were cultured for 48 hr at 37°C and 5% CO_2._ The medium was refreshed every 24 hr. Biofilms that formed on the plates were washed gently three times with sterile PBS. Then, CUR was added to the experimental groups and the corresponding concentrations of DMSO were added to the control groups, followed by incubation for 24 hr.

Crystal violet (CV) and 3‐(4,5‐dimethylthiazol‐2‐yl)‐2,5‐diphenyl tetrazolium bromide (MTT) assays were employed to verify the effects of CUR on the biomass and viability of biofilms. For CV assays, the biofilms that formed on the plates were fixed with methanol after being gently washed three times with sterile PBS. Then, the samples were washed again and stained with CV solution for 15 min. The supernatants were thoroughly discarded, and the microplates were allowed to air‐dry overnight. Finally, 200 μl of 95% ethanol was used to dissolve the CV in the microplates for 20 min. For each sample, 100 μl of the solution was analyzed on a spectrophotometer, and the absorbance at 600 nm was recorded. For MTT assays, 200 μl of 0.5 mg/ml MTT was added to the wells and incubated at 37°C for 3 hr in the dark (Li et al., 2018). Then, the supernatants were displaced by 100 μl of 100% DMSO for 10 min while protected from light. For each sample, 75 μl of the solution was analyzed on a spectrophotometer and the absorbance at 570 nm was recorded using a microplate reader.

### 2.3 Analysis of the composition of bacteria in biofilms by live–dead PCR

Live–dead PCR was used to analyze the composition of microorganisms in biofilms. Propidium monoazide (PMA) was applied to examine the quantity of live and dead microorganisms in biofilms as previously described in the literature (Sherry et al., 2016; Yasunaga et al., 2013). Because PMA is unable to permeate intact membranes of viable cells, it can only penetrate dead cells following membrane damage and bind to the DNA of dead cells following exposure to a halogen light source. This covalent bonding to DNA prevents amplification during quantitative PCR (qPCR); therefore, only live cells can be detected. No PMA‐added groups were included for each sample to determine total cells.

The biofilms were collected with a cell scraper and added to 2 ml microcentrifuge tubes with 1 ml of PBS. After resuspension, 5 μl aliquots of 10 μM PMA were added to the samples. Next, the samples were collected by centrifugation (13,201 *g*, 5 min, 4°C). Genomic DNA was extracted by a DNeasy Blood & Tissue Kit (QIAGEN) and analyzed to confirm the density and purity. Samples were analyzed using quantitative PCR (qPCR), and the Ct values were input into the formula for a standard curve to determine the quantity of microorganisms in each of the biofilms. Details were described in a previous study (Zhou et al., 2018). The primer sequences used were from previous studies in Table 1.

**Table 1:** Nucleotide sequence of primers used for PCR

Gene	Primer sequence (5′−3′)	
	Forward	Reverse
*Streptococcus mutans*	GATACATAGCCGACCTGAG	CCATTGCCGAAGATTCC (Sherry et al., 2016)^a^
*Candida albicans*	GGGTTTGCTTGAAAGACGGTA	TTGAAGATATACGTGGTGGACGTTA (Sherry et al., 2016)^a^
gtfB	ACACTTTCGGGTGGCTTG	GCTTAGATGTCACTTCGGTTG (Li et al., 2018)^a^
gtfC	CCAAAATGGTATTATGGCTGTCG	GAGTCTCTATCAAAGTAACGCAGT (Li et al., 2018)^a^
gbpB	AGCAACAGAAGCACAACCATCAG	CCACCATTACCCCAGTAGTTTCC (Li et al., 2018)^a^
comC	GACTTTAAAGAAATTAAGACTG	AAGCTTGTGTAAAACTTCTGT (Li et al., 2018)^a^
comD	CTCTGATTGACCATTCTTCTGG	CATTCTGAGTTTATGCCCCTC (Li et al., 2018)^a^
comE	CCTGAAAAGGGCAATCACCAG	GGGGCATAAACTCAGAATGTGTCG (Li et al., 2018)^a^
16S rRNA	CTTACCAGGTCTTGACATCCCG	ACCCAACATCTCACGACACGAG (Li et al., 2018)^a^
als1	TTCTCATGAATCAGCATCCACAA	CAGAATTTTCACCCATACTTGGTTTC (Alalwan et al., 2017)^a^
als3	CAACTTGGGTTATTGAAACAAAAACA	AGAAACAGAAACCCAAGAACAACCT (Alalwan et al., 2017)^a^
18S rRNA	AAACGGCTACCACATCCAAG	CCAAGCCCAAGGTTCAACTA (Barker et al., 2004)^a^

a The numbers in the brackets after the primer sequences were references.

### 2.4 The effect of CUR on the EPS of biofilms by CLSM

Confocal laser scanning microscopy was used to examine the amount of EPS in biofilms. The EPS matrix was labeled with Alexa Fluor^®^ 647‐labeled dextran conjugate, a kind of red fluorescent dye (Invitrogen Corp), at the beginning of biofilm formation and was washed gently with sterile normal saline (NS) after 72 hr (Klein et al., 2009; Yang, Liu, He, Chen, & Li, 2016). Then, SYTO9 green fluorescent dye (Molecular Probes) was applied to stain the microorganism cells for 15 min at room temperature. For image collection, the gates were set to 655–668 nm for Alexa Fluor^®^ 647‐labeled dextran conjugate and 480–500 nm for SYTO9 (Li et al., 2018). Images were collected by CLSM and analyzed by COMSTAT (Liu et al., 2017).

### 2.5 Observation of the morphology of the microorganisms in biofilms

Scanning electron microscopy (SEM) was applied to observe the structure of biofilms. The treated biofilms were collected as previously mentioned. Then, the biofilms were fixed with 2.5% glutaraldehyde overnight. The biofilms were washed with sterile PBS and dehydrated by an alcohol gradient (30%, 50%, 70%, 90%, and 100%). After that, the biofilms were treated with tert‐butanol three times. Finally, samples were dried by lyophilization and metal spraying. The samples were observed at 2,000× and 10,000× magnification by SEM.

### 2.6 Detection of gene expression in the biofilms

The biofilms were collected by centrifugation (13,201 *g*, 5 min, 4°C). Next, the total RNA was extracted by an miRNeasy Mini Kit (QIAGEN) and analyzed by a Nanodrop 2000 spectrophotometer to confirm the density and purity. Reverse transcription of the total RNA was performed. A cDNA library was constructed, and PCR was performed with reference to previous studies (Zhou et al., 2018). Table 1 shows the primer sequences (Alalwan et al., 2017; Barker et al., 2004; Li et al., 2018; Sherry et al., 2016). The results were calculated by the 2^−ΔΔCt^ method.

### 2.7 Statistical analysis

All experiments were independently performed in triplicate. GraphPad Prism version 7.0 (GraphPad Software) and SPSS 17.0 were employed to analyze the data. An unpaired *t* test was applied to analyze the data. A *p* value < .05 was set as the significance level.

## 3 RESULTS

### 3.1 Inhibition of biofilm formation by CUR

**Figure 1:**
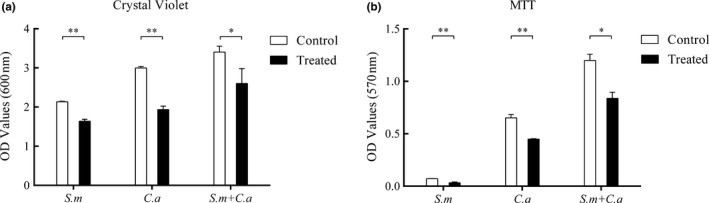
Effect of 0.5 mM CUR on mono‐ and dual‐species biofilms. Different types of mature biofilms were incubated with CUR for 24 hr. The biofilm biomass was evaluated by CV assay (a), while the viability of biofilms was evaluated by MTT assay (b). The asterisks (*) indicate significant differences (*p < .05; **p < .01). CUR decreased the biomass and viability of all treated groups

The MBEC was shown to be 0.5 mM CUR for *S. mutans* as well as *C. albicans* by assay. According to the CV and MTT assays, the biomass and viability of biofilms were greater in dual‐species biofilms despite the addition of CUR (Figure 1). After 24 hr of incubation with CUR, the biomasses of both mono‐ and dual‐species biofilms were significantly reduced compared with that of the control groups, specifically by 23% for *S. mutans*, 36% for *C. albicans*, and 24% for dual‐species biofilms (*p* < .05, Figure 1a). The viability of the biofilms decreased significantly in all treated groups (*p* < .05, Figure 1b). The viability of the *S. mutans* biofilms decreased by 54%, while those of the *C. albicans* and dual‐species biofilms decreased by 29% and 30%, respectively, relative to the control group (Figure 1b).

### 3.2 Inhibition of live/dead cells in biofilms by CUR

**Figure A1:**
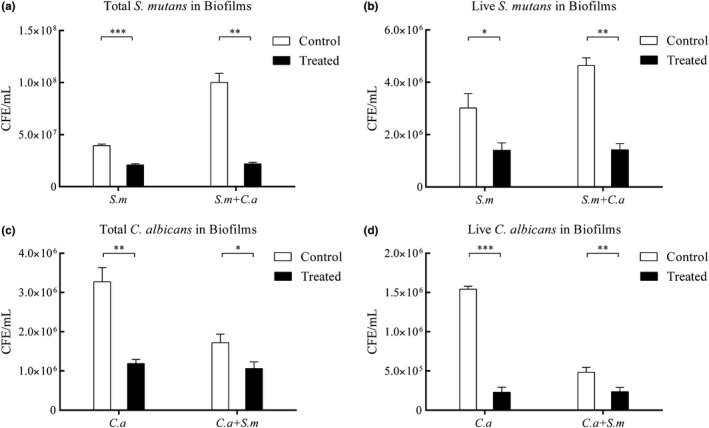
Effect of CUR on total/live microorganisms in different biofilms. Dual‐species biofilm models were grown for 48 h, and spent supernatants were replaced with fresh AS every 24 h. After mature biofilm formed, the biofilms were treated with 0.5 mM CUR for 24 h. Biofilms were assessed for total and viable composition of *C. albicans* and *S. mutans* using viability qRT‐PCR [Correction added on 21 October 2019 after first online publication: Figure S1 from Supporting Information has been moved to Appendix section as Figure A1]

Curcumin decreased total/live cells in both mono‐species and dual‐species biofilms (Figure A1), but the percent reductions with CUR in *S. mutans* and *C. albicans* biofilms were different.

**Figure 2:**
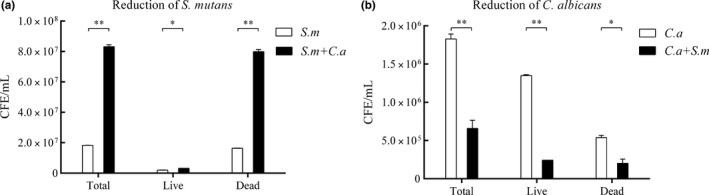
Effect of CUR on live/dead microorganisms in different types of biofilms. Dual‐species biofilm models were grown for 48 hr, and spent supernatants were replaced with fresh ASS every 24 hr. After mature biofilms formed, the biofilms were treated with 0.5 mM CUR for 24 hr. The net reduction (net reduction equals the treated group minus the control group) of *Streptococcus mutans* (a) and *Candida albicans* (b) in mono‐ and dual‐species biofilms was evaluated by species‐specific qPCR. The asterisks (*) indicate significant differences (*p < .05; **p < .01)

For the total *S. mutans* cells, CUR inhibited 79% of *S. mutans* in dual‐species biofilms while inhibiting 47% in mono‐species biofilms (*p* < .001). A similar trend was observed for live *S. mutans* cells: CUR inhibited 69% of *S. mutans* in dual‐species biofilms while inhibiting 58% in mono‐species biofilm (*p* < .001) (Figure 2a). For the total *C. albicans* cells, CUR inhibited 38% of *C. albicans* in dual‐speices biofilms while inhibiting 60% in mono‐species biofilms (*p* < .001). The same trend was observed for live *C. albicans* cells, with 52% reduction of *C. albicans* in dual‐speices biofilms and 86% inhibition in mono‐species biofilm (*p* < .001) (Figure 2b).

### 3.3 CUR‐induced EPS reduction of mono‐ and dual‐species biofilms by CLSM

**Figure 3:**
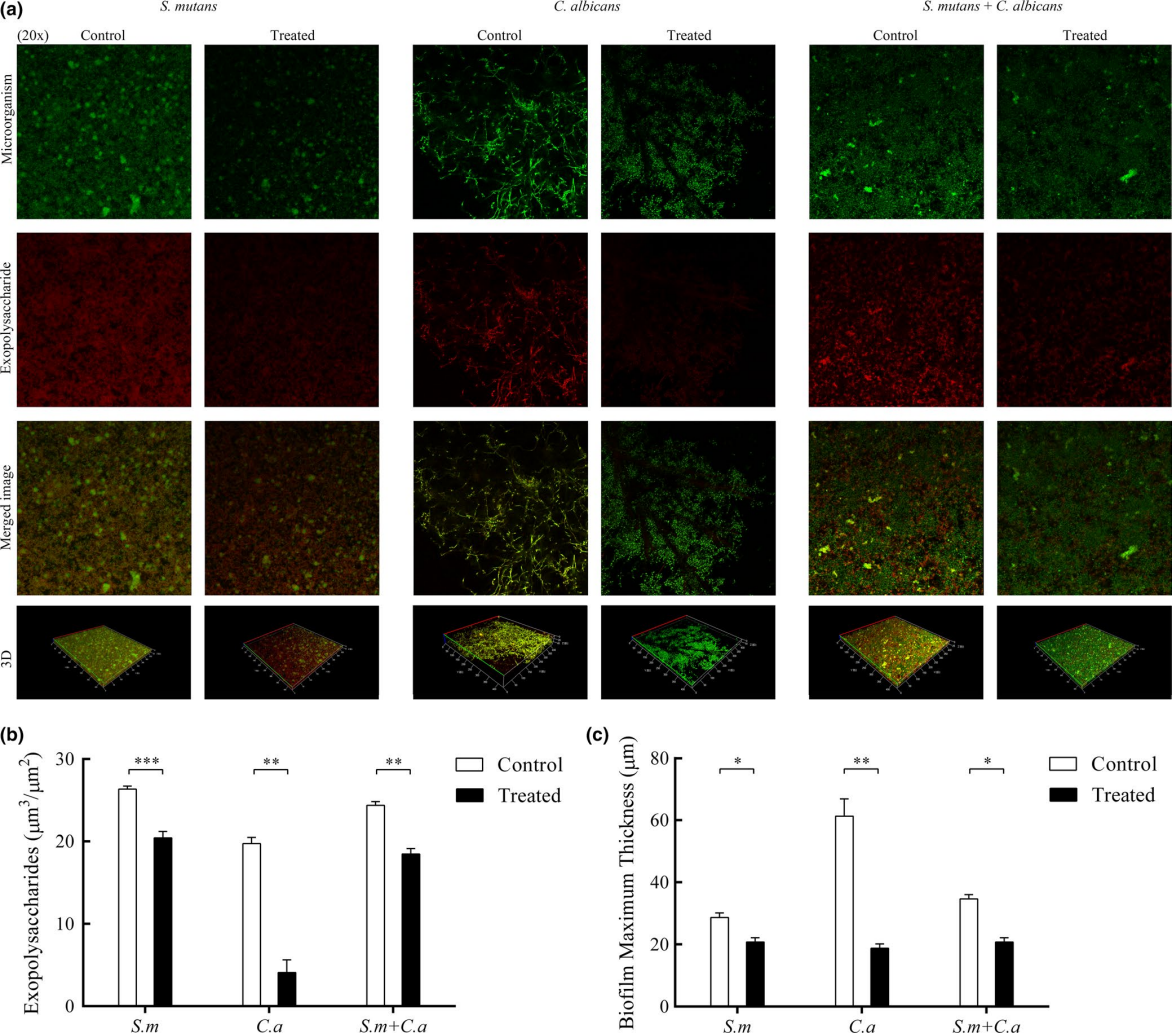
Effect of CUR on the EPS of mono‐ and dual‐species biofilms by CLSM. After incubation and staining, images of the biofilms were collected. The green channel was used for microorganism (a). The red channel was used for EPS (a). The EPS and thickness of the biofilms were quantified and compared (b, c). The asterisks (*) indicate significant differences (*p < .05; **p < .01; ***p < .001)

The amount of EPS in mono‐ and dual‐species biofilms was quantified by CLSM. The production of EPS was decreased both in mono‐ and dual‐species biofilms by CUR, as determined by CLSM images (Figure 3). The EPS in the treated group was sparser than that in the control group (Figure 3a). This result was consistent with Figure 3b, which showed a reduction in EPS in both mono‐ and dual‐species biofilms. The dual‐species group was reduced by 24% relative to the control group. *Streptococcus mutans* biofilms had a 22% reduction in EPS, and *C. albicans* biofilms had an 80% reduction in EPS. Likewise, the thickness of the biofilms in the treated groups was overtly attenuated compared with that in the control groups (Figure 3c).

### 3.4 Morphology of microorganisms in biofilms by SEM

**Figure 4:**
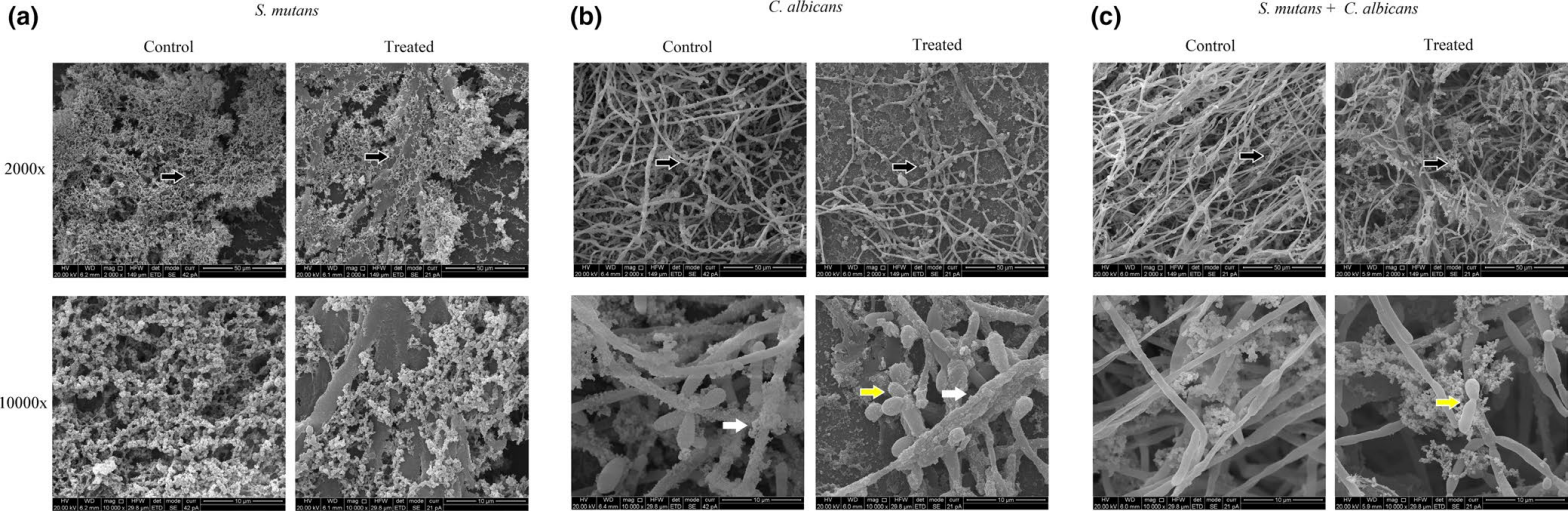
Morphological characteristics of mono‐ and dual‐species biofilms under CUR treatment. Representative SEM images of *Streptococcus mutans* (a), *Candida albicans* (b), and dual‐species biofilms (c). Each field of vision was magnified 2,000× and 10,000×. The black arrows indicate the magnified viewing area. The yellow arrows indicate the yeast. The white arrows indicate the EPS in the biofilms

Scanning electron microscopy images revealed biofilm morphologies and supported the CLSM results. In *S. mutans* mono‐species biofilms, the biofilm structures in the treated group were lost (Figure 4a). The data also showed that CUR treatment not only led to changes in biofilm structure but also caused slackening of the matrix in *C. albicans* mono‐species biofilms (Figure 4b). The structure of dual‐species biofilm seemed more disordered in the treated group than in the control group. Notably, the arrangement of hypha was altered, and less matrix was observed in the treated group of dual‐species biofilms (Figure 4c).

### 3.5 Gene expression changes in biofilms

**Figure 5:**
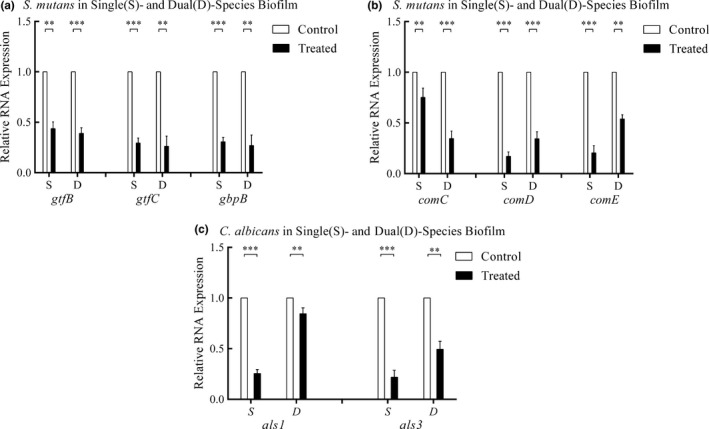
Change in gene expression in biofilms under the effects of CUR. The mRNA levels of genes in different virulence systems of *Streptococcus mutans and Candida albicans* are shown in Figure 5. Different levels of gene expression were standardized to 16 sRNA levels. (a) Relative RNA expression of *gtfs* in *S. mutans*, (b) relative RNA expression of two‐component signal transduction system in *S. mutans*, and (c) relative RNA expression of *als* in *C. albicans*. The asterisks (*) indicated significant differences (*p < .05; **p < .01; ***p < .001)

The mRNA levels of all genes tested were decreased in the CUR‐treated group relative to the control group. CUR decreased the expression of virulence‐related genes in both mono‐ and dual‐species biofilms (Figure 5).

For *S. mutans*, the relative expression levels of the virulence genes *gtfB*, *gtfC,* and *gbpB* in the mono‐species biofilms were 44%, 29%, and 31%, respectively, compared with the levels in the control groups, while in the dual‐species biofilm they were 39%, 26%, and 27%, respectively (Figure 5a). The genes of the two‐component signal transduction system, including *comC*, *comD,* and *comE,* were evaluated. The relative gene expression levels of *comC*, *comD,* and *comE* were downregulated by 25%, 83%, and 80%, respectively, in mono‐species biofilms and by 65%, 66%, and 46%, respectively, in dual‐species biofilms (Figure 5b).

In *C. albicans*, the expression levels of genes related to biofilm formation, *als1* and *als3*, were downregulated by 75% and 78%, respectively, in mono‐species biofilms and by 56% and 69%, respectively, in dual‐species biofilms (Figure 5c).

## 4 DISCUSSION

Early childhood caries has been treated using different modalities but with little success. Bacterial–fungal interactions commonly occur in the process of ECC formation. Combating this cross‐kingdom infection is a challenge in the control of ECC (Klinke et al., 2014; Yang et al., 2012). Natural products are an alternative option that results in less antibiotic resistance, and they are widely used in clinical settings (Jeon, Rosalen, Falsetta, & Koo, 2011; Nijampatnam et al., 2018; Townsley & Shank, 2017). The present study showed the value of applying CUR to address oral dual‐species biofilms.

Curcumin inhibited the biomass and viability of both mono‐ and dual‐species biofilms. A previous study reported that CUR exerted both short‐ and long‐term effects on the viability of *S. mutans* biofilms at 0.5 mM (Li et al., 2018). Alalwan et al. found that 200 μg/ml CUR (approximately equal to 0.543 mM) caused an 80% reduction in the metabolic activity of *C. albicans* biofilms (Alalwan et al., 2017). This finding is consistent with the present study. To determine the biological basis of the effect of CUR on dual‐species biofilms, live–dead PCR was conducted to assay the composition of cells in the biofilms (Sherry et al., 2016). CUR affected *S. mutans* and *C. albicans* in both dual‐ and mono‐species biofilms. However, the two microorganisms showed different responses to CUR. Many more cells of *S. mutans* were eradicated in dual‐species biofilms. The interaction between *S. mutans* and *C. albicans* enhanced the effect of CUR on *S. mutans* in dual‐species biofilms. Bacteria–fungi interactions and their relevance in health are well established (Arvanitis & Mylonakis, 2015). Dual‐species biofilms achieve higher biomass and cell numbers than mono‐species biofilms (Sztajer et al., 2014). *Streptococcus mutans‐*derived glucosyltransferase B (GtfB) itself can promote C. albicans biofilm development (Ellepola, Liu, Cao, Koo, & Seneviratne, 2017). The enhanced inhibition of *S. mutans* by CUR in dual‐species biofilms shown here is interesting.

Exopolysaccharides increases in dual‐species biofilms and enhances the cariogenic potential of biofilms (Mitchell et al., 2017). CUR influences the amount of EPS in all biofilms, which was confirmed here by CLSM. A previous study indicated that the presence of *C. albicans* augmented the production of EPS, and dual‐species biofilms accrued more biomass and harbored more viable *S. mutans* cells than mono‐species biofilms (Falsetta et al., 2014). Similarly, the presence of *Pseudomonas aeruginosa* protected *Salmonella* cells in biofilms from disinfection treatment by generating greater EPS production in dual‐species biofilms than mono‐species biofilms (Pang, Yang, & Yuk, 2017). Kim et al. (2018) found that bacteria‐derived EPS enhanced antifungal drug tolerance in a cross‐kingdom oral biofilm. In mono‐species biofilms, we found that CUR disrupted EPS secretion not only from *S. mutans* but also from *C. albicans* biofilms. It is likely that CUR affects EPS production, either by fungi or bacteria, to disrupt dual‐species biofilms.

*Streptococcus mutans* extracellular glucosyltransferases (Gtfs), particularly GtfB and GtfC, synthesize predominantly water‐insoluble glucans, which contribute to the structural scaffold of biofilms (Bowen & Koo, 2011). A previous study showed that deletion of *gtfB* and *gtfC* significantly disrupted biofilm formation, which corresponded with the results in this study (Ooshima et al., 2001). In our study, *gtfB* and *gtfC* were inhibited by CUR relative to the control group, and a reduction in EPS and structural looseness were observed in biofilms treated with CUR. Quorum sensing (QS) systems have a key role in coordinating biofilm formation and activating virulence factors in many bacteria and are considered an enticing target for fighting biofilm infection (Suntharalingam & Cvitkovitch, 2005; Worthington, Richards, & Melander, 2012). Among these, the competence‐stimulating peptide‐QS (CSP‐QS) system is the most common intraspecific example, including a competence‐stimulating peptide (encoded by *comC*), a histidine kinase sensor protein (encoded by *comD*), and a cognate response regulator (encoded by *comE*) (Suntharalingam & Cvitkovitch, 2005; Yue et al., 2018). A previous study found that in the presence of chlorhexidine, the upregulation of immB in a *comC* mutant was largely inhibited compared with that in the wild‐type strain, implying that the function of the QS system is one of the mechanisms regulating *S. mutans* antimicrobial sensitivity (Wang, Liu, Huo, & Ling, 2013). In the present study, the expression of *comC*, *comD,* and *comE* was suppressed by CUR in all biofilms. The results indicated that CUR inhibition occurs through the QS system.

The agglutinin‐like sequence (Als) family plays an important role in adhesion and aggregation with other substances, and it is a key element for *C. albicans* in biofilm formation (Hoyer & Cota, 2016)*.* The gene expression of *als1* and *als3* decreased sharply after CUR treatment. This may have affected the function of the Als family and alleviated the interaction of *C. albicans* with bacteria. Lee et al. suggested that CUR exerted antifungal activity by inducing disruption of the fungal plasma membrane (Lee & Lee, 2014). CUR most likely hampers the Als family of *C. albicans* to exert antibacterial effects on *S. mutans* in dual‐species biofilms.

## 5 CONCLUSIONS

Curcumin induces effects on *S. mutans* and *C. albicans*, such as their EPS formation, through different biological systems. *Candida albicans* is less sensitive to CUR in fungi–bacteria interkingdom biofilms.

## CONFLICT OF INTEREST

None declared.

## AUTHOR CONTRIBUTION

Xinlong Li and Luoping Yin participated in the study design, carried out the experimental studies on biofilms, and performed statistical analysis. Gordon Ramage helped to draft the manuscript. Binchun Li, Ye Tao, and Qinghui Zhi participated in the study design and assisted with statistical support. Huancai Lin and Yan Zhou conceived the study, participated in the study design and data analysis, and were responsible for writing and submitting the final manuscript. All authors read and approved the manuscript.

## ETHICAL APPROVAL

None required.

## Data Availability

All data generated or analyzed during this study are included in this published article.
